# Association of Heme Oxygenase 1 with Lung Protection in Malaria-Associated ALI/ARDS

**DOI:** 10.1155/2016/4158698

**Published:** 2016-11-15

**Authors:** Marcelo L. M. Pereira, Luana S. Ortolan, Michelle K. Sercundes, Daniela Debone, Oscar Murillo, Flávia A. Lima, Claudio R. F. Marinho, Sabrina Epiphanio

**Affiliations:** ^1^Departamento de Imunologia, Instituto de Ciências Biomédicas, Universidade de São Paulo, São Paulo, SP, Brazil; ^2^Departamento de Análises Clínicas e Toxicológicas, Faculdade de Ciências Farmacêuticas, Universidade de São Paulo, São Paulo, SP, Brazil; ^3^Departamento de Parasitologia, Instituto de Ciências Biomédicas, Universidade de São Paulo, São Paulo, SP, Brazil

## Abstract

Malaria is a serious disease, caused by the parasite of the genus* Plasmodium*, which was responsible for 440,000 deaths in 2015. Acute lung injury/acute respiratory distress syndrome (ALI/ARDS) is one of the main clinical complications in severe malaria. The murine model DBA/2 reproduces the clinical signs of ALI/ARDS in humans, when infected with* Plasmodium berghei* ANKA. High levels of HO-1 were reported in cases of severe malaria. Our data indicated that the HO-1 mRNA and protein expression are increased in mice that develop malaria-associated ALI/ARDS (MA-ALI/ARDS). Additionally, the hemin, a HO-1 inducing drug, prevented mice from developing MA-ALI/ARDS when administered prior to the development of MA-ALI/ARDS in this model. Also, hemin treatment showed an amelioration of respiratory parameters in mice, high VEGF levels in the sera, and a decrease in vascular permeability in the lung, which are signs of ALI/ARDS. Therefore, the induction of HO-1 before the development of MA-ALI/ARDS could be protective. However, the increased expression of HO-1 on the onset of MA-ALI/ARDS development may represent an effort to revert the phenotype of this syndrome by the host. We therefore confirm that HO-1 inducing drugs could be used for prevention of MA-ALI/ARDS in humans.

## 1. Introduction

Malaria is a serious disease caused by the* Plasmodium* parasite and transmitted through the bite of the* Anopheles* mosquito. It is estimated that there were about 214 million cases of malaria in 2015, resulting in approximately 440,000 deaths, most of which originating from sub-Saharan Africa and in children under 5 years of age [1].

Malaria is characterized by signs and symptoms such as severe anemia, fever, vomiting, and fatigue [2]. During the symptomatic phase of malaria several clinical complications can occur and are defined as severe malaria. These complications are anemia, cerebral malaria, placental malaria, and acute lung injury/acute respiratory distress syndrome (ALI/ARDS) [3]. The ALI/ARDS has been diagnosed in patients suffering from malaria caused by all the species that cause disease in humans, including* P. knowlesi* [4]; however it is more common in* P. falciparum* and* P. vivax* malaria [5].

ALI/ARDS is characterized by high morbidity and, although more common in adults, also affects children and pregnant woman. The most common manifestations of this syndrome are noncardiogenic pulmonary edema, increased phagocytic activity, dyspnea, reduction in the capacity of gas exchange, and increased levels of inflammatory mediators [6]. ALI/ARDS is most commonly caused by bacteria, sepsis, viral pneumonia, gastric aspiration, severe trauma, adverse drug reactions, and fungal or parasitic infections of the lung [7]. The mechanisms that are critical in the initial and later stages of ALI/ARDS are not well defined [8]. However, it is known that the presence of intravascular fluid in the lungs, due to increased permeability of the alveolar capillary membrane, is the key pathophysiological mechanism of ALI/ARDS [9].

Multiple factors are possibly involved in the increased vascular permeability, such as endothelium injury, increased levels of proinflammatory cytokines such as TNF-*α* (tumor necrosis factor alpha), interleukin 1 (IL-1), or IL-6 and IL-8, and endovascular occlusion associated with the accumulation of erythrocytes with reduced deformability, leukocytes, and platelets [6, 9].

Different mouse models have been developed for the study of MA-ALI/ARDS, showing similar aspects to human ALI/ARDS [10–13]. DBA/2 strain mice develop ALI/ARDS when infected with the parasite* Plasmodium berghei* ANKA (PbA) [10]. In this model, an average of 50% of the mice that die between days 7 and 12 after infection have dyspnea, hypoxemia, and reduced respiratory rate. Postmortem studies revealed that these mice had pleural effusion, containing cells such as neutrophils, lymphocytes, monocytes, and macrophages [10, 14]. Furthermore, the vascular endothelial growth factor (VEGF) has been identified as critical in increased pulmonary vascular permeability, a hallmark of ALI/ARDS [10, 15].

The protective role of HO-1 enzyme and carbon monoxide (CO) in experimental severe malaria episodes has been demonstrated in animal models [10, 15, 16]. Additionally, HO-1 was found to be increased in peripheral blood leukocytes, plasma, tissue macrophages, and monocytes of humans with severe malaria [17, 18]. HO-1 is an enzyme encoded by* hmox-1* gene and is considered “protective” due to its anti-inflammatory, antiapoptotic, and antiproliferative actions in different cell types, including endothelial cells [19]. This enzyme participates in the free heme degradation, generating equimolar amounts of CO, iron, and biliverdin and plays a protective role in modulating tissue response to injury in various organs, including the lung [20–22]. The malaria infection leads to the release of reactive oxygen species and free heme, harmful to the endothelial cells of the host. When exposed to free heme, host cells increase expression of HO-1 [23]. HO-1 catabolizes free heme into iron, biliverdin, and CO, which are less toxic to the cells. Therefore, some studies have shown that inducers of HO-1 such as hemin and cobalt protoporphyrin IX (CoPPIX) protected mice infected with malaria, or suffering from other diseases such as polymicrobial sepsis, from developing ALI/ARDS [16, 24, 25]. Additionally, it was observed in previous publications that HO-1 inducers are protective against experimental cerebral malaria [16] and that treatment of DBA/2 mice with a CO-releasing molecule is protective against MA-ALI/ARDS [15]. Meanwhile, in this study, it was observed, for the first time that HO-1 expression is increased in PbA infected DBA/2 mice lungs which develop ALI/ARDS and that the induction of HO-1 protects these mice from developing ALI/ARDS.

## 2. Materials and Methods

### 2.1. Mice, Parasites, and Infection

Six- to ten-week-old male DBA/2 mice were bred under pathogen-free conditions in isogenic mouse facilities, at the Biomedical Sciences Institute of the University of São Paulo. In all experiments, the welfare of the animals was taken into consideration. Mice had* ad libitum* access to water and food (Nuvilab CR-1, Quintia S/A, São Paulo, Brazil). Mice were intraperitoneally infected with 1 × 10^6^ *Plasmodium berghei* ANKA (clone 1.49L) infected red blood cells (iRBCs), as previously described [10]. Parasitemia levels were monitored daily using Giemsa-stained peripheral blood smears. All experiments were performed in accordance with the ethical guidelines for experiments with mice, and the protocols were approved by the Animal Health Committee of the Biomedical Sciences Institute of the University of São Paulo (CEUA number 146, page 136, book 2). The guidelines for animal use and care were based on the standards established by The Brazilian College of Animal Experimentation (COBEA). All efforts were made to prevent undue stress or pain to the mice. Twenty days after infection, all surviving animals were euthanized. The mice were euthanized with an anesthetic overdose of ketamine (150 mg/kg) (Vetbrands, Brazil) and xylazine (15 mg/kg) (Syntec, Brazil), and consciousness was checked by testing the pedal reflex, heartbeats, and breathing movements.

### 2.2. Histological Evaluations

Necropsy was performed in mice dying naturally from malaria or mice euthanized on the 20th day after infection (DAI) to complete the experiment and to avoid animal suffering. The lungs were collected and fixed in buffered 10% formalin for 24 hours and 70% alcohol for 24–48 hours and then embedded in paraffin, sectioned at 5 *μ*m onto slides, and stained with hematoxylin-eosin (HE) according to standard protocol. Histopathological analyses were performed under a Axio Imager M2 (Zeiss) microscope using the Axio Cam HRc (Zeiss) and the software Axio Vision, version 4.9.1.0. In order to determine the alveolar area percentage, 20 pictures per lung HE section were taken and the software Gimp, version 2.8.16 (https://www.gimp.org/) was used to determine the alveolar area in pixels of each picture. The percentage of alveolar area relatively to total area of the pictures were determined in ALI/ARDS versus HP and in hemin treated versus nontreated (saline) mice.

### 2.3. Determination of Respiratory Pattern

Respiratory patterns (respiratory frequency (RF) and enhanced pause (Penh)) were monitored on the 7th DAI by placing the mice in the plethysmography chamber (WBP, Buxco Electronics, Wilmington, North Carolina, USA) for 10 minutes (basal level) as described before [14]. The data were collected using Biosystems XA software and included the RF (breaths/minute) and variables to calculate the Penh, a theoretical variable that correlates with both pulmonary resistance and intrapleural pressure [26]. The Penh is calculated by the following equation [27]:

(1) Penh=peak  expiratory  heightpeak  inspiratory  height×expiratory  timerelaxation  time−1.

### 2.4. Identifying ALI/ARDS in Mice before Death

Identification of ALI/ARDS in mice before death was done as described before [14]. In short, we used two groups of PbA infected mice (10–12 mice per group): the survival group and the euthanized group in which the mice were euthanized on the 7th DAI. In the survival group, in any mouse that died between the 7th and 12th DAI showing pleural effusion or red and congested lungs at necropsy, the cause of death was attributed to ALI/ARDS. In contrast, in mice without pleural effusion that died after 13th DAI with pale lungs and high levels of parasitemia, the cause of death was attributed to hyperparasitemia (HP) and, consequently, anemia. In the euthanized group, mice were classified as having been likely to die of ALI/ARDS or HP, by comparing their respiratory patterns and parasitemia levels with the survival group, in which the* causa mortis* was known, as previously published [14].

### 2.5. HO-1 Immunohistochemistry

To perform the immunohistochemistry of HO-1, slides containing the tissue sections were placed in an incubator at 60°C for 20 minutes. Then they were incubated in xylene twice for 15 min at 60°C and afterwards passed in absolute ethanol, in 95% alcohol, in 70% alcohol, in distilled water, and finally in 1x PBS pH 7.2 to 7.4. For the antigen retrieval, the slides were incubated in sodium citrate buffer pH 6 for 45 minutes at 95°C. Endogenous peroxidase was blocked with 3% hydrogen peroxide for 15 minutes, twice, at room temperature and protected from light. The tissue was probed with rabbit polyclonal to HO-1 (1 : 1000) antibody (Abcam, ab13243) overnight at 4°C and then the kit REVEAL mouse/rabbit (SPRING-Code SPD-015) was used in accordance with the manufacturer's instructions. The quantification of HO-1 in lung tissue was done by calculating the marked area in the entire tissue section. The calculation was done in ImageJ (version 1.50b) software (https://imagej.nih.gov/ij/), using the plugin IHC toolbox (http://rsb.info.nih.gov/ij/plugins/ihc-toolbox/index.html) [28].

### 2.6. Western Blot

Fresh frozen mice lung tissues were sonicated and homogenized in ice using the Radio-Immunoprecipitation Assay (RIPA) buffer composed of 50 mM Tris-HCl pH 8.0, 150 mM NaCl, 0.5% sodium deoxycholate, 0.1% sodium dodecyl sulphate (SDS), 1 mM sodium orthovanadate, 1 mM NaF, and a protease inhibitor tablet. The samples were analyzed for protein content using a Bradford protein assay (BioRad) according to manufacturer's instructions. Each sample was quantified, and then 9 *μ*g of protein was loaded onto a 12% Tris-glycine SDS-polyacrylamide gel, according to the manufacturer's protocol (BioRad). The gel was transferred to a PVDF membrane by electrophoresis at 30 V for 16 h. The membrane was blocked in PBS with 0.1% Tween 20 (PBS-T) and 10% nonfat milk at room temperature for 2 h. All antibodies were diluted in the same buffer (PBS-T) with 1% nonfat milk.

The membrane was then probed with rabbit polyclonal HO-1 antibody (Abcam, ab13243, 1 : 20,000) and then incubated for 1 h at room temperature. After incubation, the membrane was washed five times with PBS-T and incubated with horseradish peroxidase–conjugated goat anti-rabbit IgG (Millipore, ap307p, 1 : 20,000) for 1 h at room temperature. After washing five times with PBS-T, an ECL system (Clarity Western ECL Blotting Substrate, Biorad) was used for detection of the proteins in a ChemiDoc XRS+ System (Biorad). The HO-1 expression was calculated by densitometry, in the software ImageJ (version 1.50b) of the HO-1 bands in the immunoblot relative to the housekeeping protein *β*-actin (rabbit monoclonal to *β*-actin, Novus biologicals, NB600-501, 1 : 500,000).

### 2.7. HO-1 and Bilirubin Quantification by ELISA

On the 7th DAI, mice were anesthetized, and their serum was collected by cardiac puncture. ELISAs kits were used to quantify HO-1 levels in serum and macerated lung tissue (Enzo Lifesciences, ADI-960-071) and bilirubin levels (an indirect measure of HO-1 activity) (Biomatik, EKU08395) according to the manufacturer's instructions. The HO-1 level of the ALI/ARDS and HP mice was expressed as fold increase in relation to noninfected mice. The bilirubin values were presented in logarithm of the concentration in micrograms per milliliter.

### 2.8. Quantitative RT-PCR

Extraction of total RNA from lungs of mice was performed using RNeasy Mini Kit (Qiagen), according to the manufacturer's instructions. Noninfected mice were used as controls and as baseline levels. One microgram of total RNA was reverse-transcribed to single-strand cDNA using the First-Strand cDNA Synthesis Kit (Roche) AMV Reverse Transcriptase protocol (Roche Applied Science). HO-1 transcripts in the cDNA obtained from the reverse transcriptase reaction were quantified by real-time quantitative fluorogenic PCR performed in the 7500 Fast Instrument (Applied Biosystems). SYBR Green PCR Master Mix (Applied Biosystems) was used to quantify gene expression according to the manufacturer's instructions. The gene expression was normalized by the housekeeping gene hypoxanthine guanine phosphoribosyltransferase (HPRT) and using the relative quantification method 2^(−ΔΔCT)^ as described before [29]. The primers used were as follows: HO-1: 5′-TCTCAGGGGGTCAGGTC-3′ (forward) and 5′-GGAGCGGTGTCTGGGATG-3′ (reverse); IFN-*γ*: 5′-CACACTGCATCTTGGCTTTG-3′ (forward) and 5′-TCTGGCTCTGCAGGATTTTC-3′; HPRT: 5′-TGCTCGAGATGTGATGAAGG-3′ (forward) and 5′-TCCCCTGTTGACTGGTCATT-3′ (reverse).

### 2.9. HO-1 Induction by Hemin

Hemin (Sigma-Aldrich) was diluted in 0.2 M NaOH to a final concentration of 5 mM and pH 7.4. Hemin was administered in two doses: the first was two days before the infection and another at 4th DAI in single doses of 17 mg/kg or with saline solution (control group), intraperitoneally. To measure parasitemias and survival rate of hemin treated and saline treated mice, they were kept alive until the 20th DAI. HO-1 induction by hemin was also used to measure lung vascular permeability and VEGF and cytokine serum levels.

### 2.10. Lung Vascular Permeability

To investigate lung vascular permeability on the 7th DAI, the hemin treated or saline treated infected mice and noninfected mice were injected intravenously with 0.2 mL of 1% Evans Blue (Sigma). The mice were euthanized 45 minutes later, and the lungs were weighed and placed in 2 mL of formamide (Merck) for 48 hours at 37°C. The absorbance of the formamide was then measured at *λ*620 nm. The amount of Evans Blue staining per gram of lung tissue was calculated from a standard curve. The lung permeability was expressed as fold increase in relation to the noninfected mice.

### 2.11. VEGF Quantification in Serum

On the 7th DAI, hemin treated or saline treated infected mice and noninfected mice were anesthetized, and their serum was collected by cardiac puncture. An ELISA kit (R&D Systems) was used to quantify VEGF levels in the serum according to the manufacturer's instructions. The VEGF level was expressed as fold increase in relation to that of the noninfected mice.

### 2.12. Cytokine Levels Measurement

The cytokine levels were measured in serum from the hemin treated and saline treated DBA/2 mice. Mice Inflammation CBA Cytokine Kit (Cytometric Bead Array, Becton-Dickinson) was used to measure interferon, IFN-*γ*, tumor necrosis factor, TNF-*α*, and IL- and IL-10 levels. This kit was used for dosing cytokines from lung tissue lysates using flow cytometer (BD FACS Calibur system, Becton-Dickinson) and using the software Cell Quest Pro, version 5.2. The individual standard curve range for a given cytokine was determined according to manufacturer instructions. The data was analyzed using FlowJo, version 10.0.7, software.

### 2.13. Isolation of the Primary Microvascular Lung Endothelial Cells

The primary microvascular lung endothelial cells (PMLEC) were obtained from DBA/2 mice, according to what was described before [30]. After euthanasia, the animal's body was disinfected with iodine alcohol. Then all blood was taken from the mice by cutting the carotid artery. In a laminar flow chamber, the lung tissue was cut into fragments of approximately 1 mm^2^ which were distributed among 6-well polystyrene plates. After being supplemented, DMEM culture medium (20% serum fetal bovine (FBS) and antibiotics) was added to each well. The plate was then incubated at 37°C and 5% CO_2_ for 72 h. After this period, the tissue fragments were removed and 50% of the medium was replaced. After 7 days of incubation, the cells were removed with trypsin 0.25% (EDTA, Gibco) for 15 min and replaced in a culture flask of 75 cm^2^. The trypsinization procedure was repeated every 5 to 7 days. Finally, the cells were cultured for 15 to 20 days (3rd and 4th passage) until being used in the following trials. The isolated PMLEC were characterized by immunofluorescence with the antibodies anti-VWF, anti-VCAM, anti-ACE, anti-CD62E, anti-eNOS, anti-CD31, and anti-VE-cadherin.

### 2.14. Identification of Actin Microfilaments by Immunofluorescent and Morphometric Analysis of the Opening of Interendothelial Junctions

To analyze the area of opening of interendothelial junctions (OIJ), the actin filaments of PMLEC were marked. In order to achieve that, the lung endothelial cells were plated in 24 well plates (7 × 10^4^ cells/well), adhered to gelatin on glass coverslips, and maintained at 37°C and 5% CO_2_. The cells were stimulated with PbA lysate for 3 h, after incubation with hemin (5, 10, and 20 *μ*M during 24 h), or solely with DMEM culture medium, supplemented with 20% FBS, in triplicate. Subsequently, the cells were fixed with 3.7% formaldehyde, permeabilized with acetone at −20°C, and blocked with bovine serum albumin solution (1% BSA). Actin was marked with Texas Red Phalloidin (Life Technologies) by 20 minutes. The cell nuclei were marked with Hoechst (H33342, Life Technologies).

Each slide, with fully confluent cells, was chosen randomly and ten to twenty pictures were taken and scanned in a “zig-zag” way, from top to bottom. The images were acquired in the fluorescence Axio Imager M2 (Zeiss) microscope using the Axio Cam HRc (Zeiss) and the software Axio Vision, version 4.9.1.0. The total area of OIJ was measured in each picture using the software Gimp, version 2.8.16 (https://www.gimp.org/).

### 2.15. Measure of Primary Microvascular Lung Endothelial Cells Permeability

The increased lung vascular permeability was analyzed in PMLEC plated on inserts of permeable membranes with pores of 0.4 *μ*M (Transwell® Corning), pretreated with gelatin and coupled in 24-well polystyrene plates at a concentration of 2.2 × 10^4^ cells per insert and maintained in DMEM culture at 37°C. After 96 hours, until the cells reach confluency, the extract was applied for 3 h after incubation with hemin (20 *μ*M during 24 h) or solely with 20% FBS supplemented DMEM culture medium. Subsequently, the culture medium was replaced by Hank's balanced salt solution and in the upper compartment of each insert (in contact with the cells); 200 *μ*L of Evans Blue was incubated at a concentration of 2 mg/mL at 37°C. After 30 min, the liquid from the lower compartment was collected and analyzed in a spectrophotometer at a wavelength of 650 nm (NanoDrop 2000, Thermo Scientific). Finally, the concentration of Evans Blue in each sample was determined from a standard curve with previously known concentrations of Evans Blue (0.2 mg/mL to 0.0031 mg/mL).

### 2.16. Statistical Analysis

The data were analyzed by D'Agostino-Pearson normality test. Nonparametric variables were compared using Mann–Whitney test and Kruskal–Wallis test with Dunn's multiple comparisons test. The unpaired* t*-test and one-way ANOVA with Bonferroni multiple comparison test were used for parametric variables. Statistical analyses were performed in GraphPad Prism version 5.0 (http://www.graphpad.com/scientific-software/prism/), including assessments of sensitivity and specificity. To establish cut-off from data, ROC curves were generated using the results of the control group in MedCalc version 8.2.1.0 (https://www.medcalc.org/). Survival curves were analyzed by Log-rank test. Data in graphs is presented representing means and SEM.

## 3. Results

### 3.1. *P. berghei* ANKA Infection of DBA/2 Mice Leads to the Development of ALI/ARDS

The development of ALI/ARDS in the DBA/2 mouse model occurred between the 7th and the 12th DAI (Figures S1A and S1B in Supplementary Material, available online at http://dx.doi.org/10.1155/2016/4158698), during which the mice have died presenting at necropsy reddish lungs, pleural effusion, and histologic changes such as congestion, alveolar edema and hemorrhage, inflammatory infiltrate, and damage to the alveolar wall (Figure S1C). Mice that survived after the 12th DAI died on the 20th DAI or were euthanized at the same day, showing grayish tone lungs, splenomegaly, and no pleural effusion, interstitial chronic pneumonia, and malarial pigment in the lung tissue (Figure S1C). These two phenotypes were described in detail previously by our group [10, 14]. Noninfected mice showed light pink lungs, and no liquid inside of the thoracic cavity was detected (Figure S1C). These changes in lung histology were consistent with the percentage of alveolar area; they were significantly lower in ALI/ARDS than in HP mice (Figure S1D).

### 3.2. The Expression of HO-1 Is Higher in ALI/ARDS-Developing Mice Compared to HP-Developing Mice

By using the predictive model to identify ALI/ARDS or HP in mice before death [14], the expression of HO-1 in the development of ALI/ARDS was determined in the serum and lungs of infected DBA/2 mice and compared to noninfected mice.

**Figure 1:**
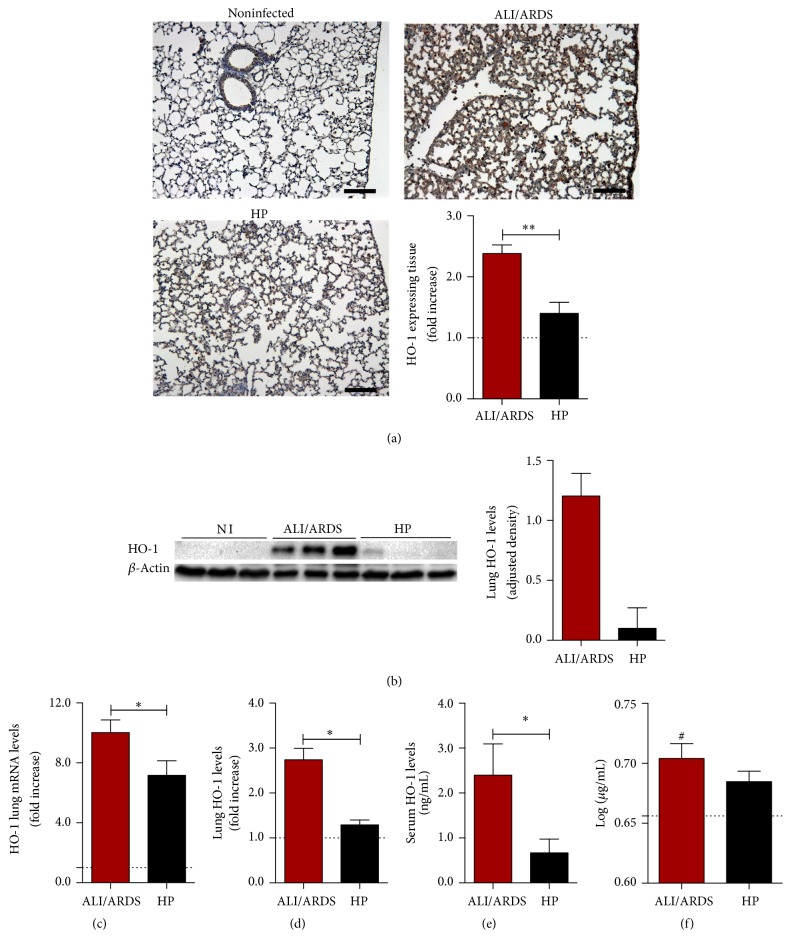
The expression of HO-1 is higher in ALI/ARDS-developing mice compared to HP-developing mice. (a) Representative images of lung sections subject to immunohistochemistry for detection of HO-1 protein (brown), counterstained with hematoxylin. The graph represents the quantification of protein expression of HO-1 by immunohistochemistry on the 7th day after infection (DAI). Dashed line represents the average of values from noninfected mice. (Mann–Whitney test, *n* = 10, ^*∗∗*^ *p* ≤ 0.01). (b) Immunoblot of HO-1 and beta actin control (left). Protein levels of HO-1 measured by immunoblot densitometry (right). Values are expressed in HO-1 band densities adjusted by the beta actin control. (c) Expression of HO-1 mRNA levels in lungs of ALI/ARDS-developing mice and HP-developing mice (unpaired* t*-test, *n* = 28, ^*∗*^ *p* ≤ 0.05). (d) Values of HO-1 in lung cell lysates of ALI/ARDS versus HP-developing mice (Mann–Whitney test, *n* = 8, ^*∗*^ *p* ≤ 0.05). (e) Protein levels of HO-1 in the serum of ALI/ARDS-developing mice and HP-developing mice (Mann–Whitney test, *n* = 9, ^*∗*^ *p* ≤ 0.05). (f) Bilirubin levels in the serum of ALI/ARDS and HP infected mice. Bilirubin levels are significantly higher in ALI/ARDS than in noninfected mice (one-way ANOVA with Bonferroni's multiple comparison test *n* = 38, ^#^ *p* ≤ 0.05). The dashed lines represent the average values of noninfected mice (minimum *n* = 3). In graphs ((b) and (e)) the values of noninfected mice were equal or less than 0. In graphs with fold increase, the values of ALI/ARDS-developing and HP-developing mice are compared to the average values of noninfected mice.

In order to observe the protein expression of HO-1, immunohistochemistry sections of lung tissue of DBA/2 mice were performed. The expression of HO-1 was higher in lungs of ALI/ARDS-developing mice than in HP-developing mice and noninfected mice. Quantification of immunohistochemical stain of HO-1 in the lung was performed by image analysis (Figure 1(a)).

HO-1 protein levels (Figure 1(b)) and HO-1 mRNA expression (Figure 1(c)), measured by western blot and qRT-PCR, respectively, in the mice lungs, were higher in ALI/ARDS-developing mice compared to HP-developing mice on the 7th DAI. Also, HO-1 levels in the lung tissue cell lysate and in the sera were higher in ALI/ARDS mice than in HP mice, as determined by ELISA (Figures 1(d) and 1(e)).

In addition, we checked the activity of HO-1 measuring bilirubin in PbA infected and noninfected mice by ELISA, on the 7th DAI. The levels of bilirubin were significantly higher in ALI/ARDS-developing mice than in noninfected mice (Figure 1(f)). However, the levels of bilirubin were not significantly different between ALI/ARDS and HP mice.

### 3.3. Induction of HO-1 Protects* Plasmodium berghei*-Infected DBA/2 Mice from ALI/ARDS

Mice were treated with 17 mg/kg of hemin, an inductor of HO-1, on the second day before PbA infection and on 4th DAI. The efficiency of induction of HO-1 protocol was checked and confirmed, showing that hemin treatment increased mRNA and protein levels of HO-1 in the lungs and in the serum of noninfected animals (Figures S2A and S2B, resp.), which confirms it as an inducer of HO-1.

**Figure 2:**
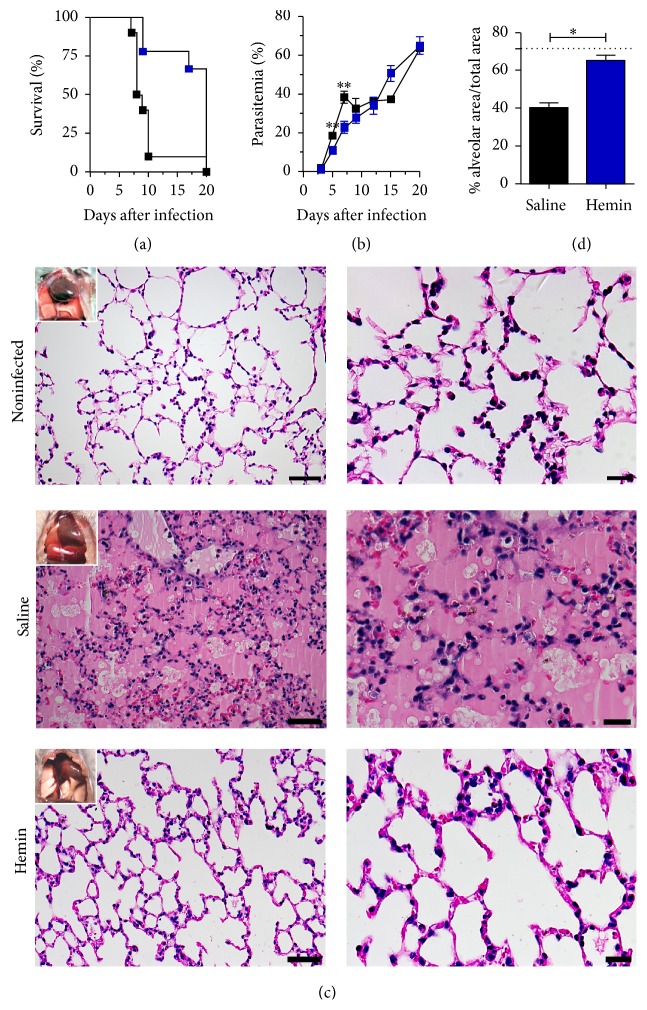
Hemin treatment protects* Plasmodium berghei*-infected DBA/2 mice from ALI/ARDS. Mice were treated with hemin on days 2 and 4 after infection. (a) Parasitemias of mice treated with hemin or saline (unpaired* t*-test, *n* = 19, ^*∗∗*^ *p* ≤ 0.01). (b) Survival curve of mice treated with hemin or saline (log-rank test, *n* = 19, *p* ≤ 0.01). (c) Representative figures of necropsies and histological lung sections of noninfected mice (top); hemin treated mice, on the 7th day after infection (middle); and saline treated mice (bottom). Scale bars: left column: 50 *μ*m; right column: 20 *μ*m; (d) alveolar area percentage in hemin treated versus saline treated mice (Mann–Whitney test, *n* = 8, ^*∗*^ *p* ≤ 0.05).

The hemin treatment increased the survival with most of the mice alive after the 12th DAI without developing ALI/ARDS (Figure 2(a)). Additionally, the parasitemia was significantly different between the 5th and the 7th DAI (Figure 2(b)).

Mice treated with saline (control) and sacrificed on 7th DAI presented necropsy reddish lungs, pleural effusion, congestion, alveolar edema and hemorrhage, inflammatory infiltrate, and damage to the alveolar wall (Figure 2(c)), similarly to what was observed in mice that developed ALI/ARDS (Figure S1) [10, 14]. On the other hand, hemin treated mice showed a phenotype similar to noninfected mice: light pink lungs, and no liquid inside of the thoracic cavity was detected (Figure 2(c)). As it was observed in ALI/ARDS versus HP mice, saline treated mice also had significantly less percentage of alveolar area than hemin treated mice (Figure 2(d)).

### 3.4. Induction of HO-1 Improves* Plasmodium berghei*-Infected DBA/2 Respiratory Parameters and Lowers Inflammatory Cytokines Levels

**Figure 3:**
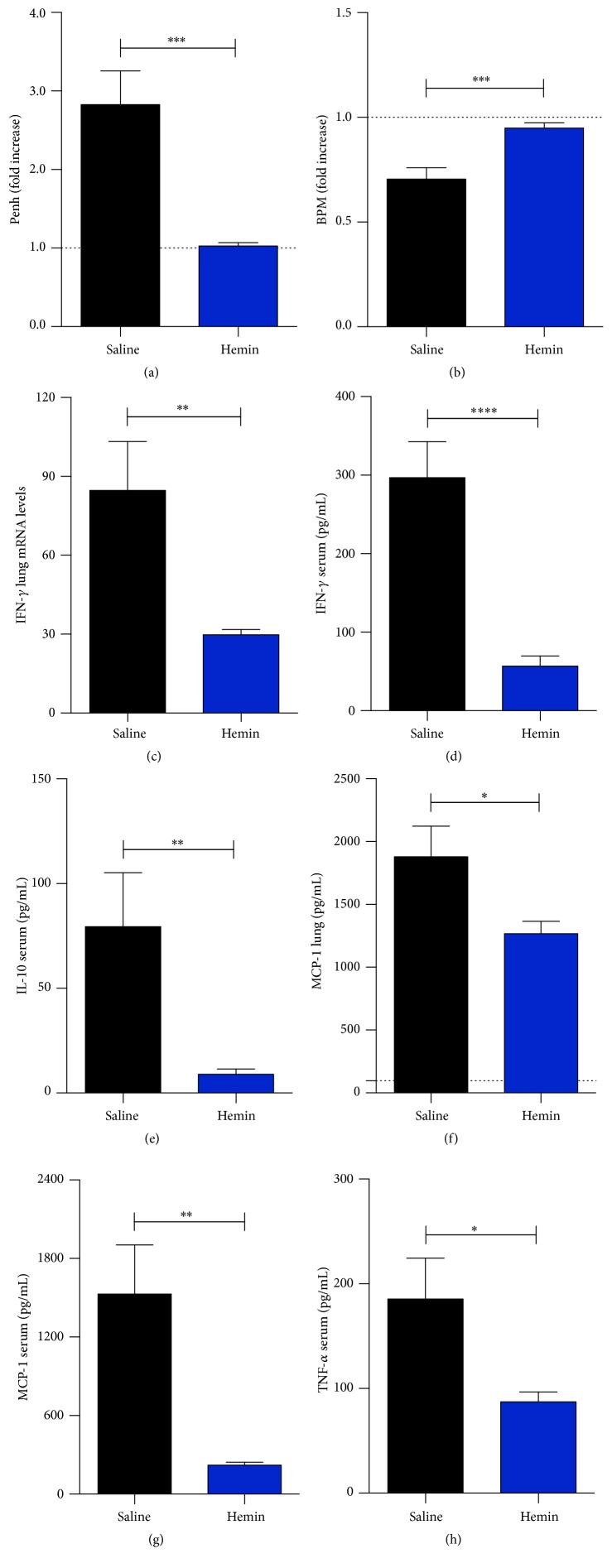
Induction of HO-1 improves* Plasmodium berghei*-infected DBA/2 respiratory parameters and lowers inflammatory cytokines levels. Mice were treated with hemin on days 2 and 4 after infection. ((a) and (b)) Respiratory pause and respiratory frequency of the animals treated and untreated after 7th DAI (unpaired* t*-test, *n* = 20, ^*∗∗∗*^ *p* ≤ 0.001). (c) IFN-*γ* quantitative RT-PCR assay of mice lung tissue (unpaired* t*-test, *n* = 20, ^*∗∗*^ *p* ≤ 0.01). ((d) to (h)) Protein levels of the cytokines IFN-*γ*, IL-10, MCP-1, and TNF-*α* in the serum and lung determined by CBA (unpaired* t*-test, *n* = 20, ^*∗*^ *p* ≤ 0.05, ^*∗∗*^ *p* ≤ 0.01, and ^*∗∗∗∗*^ *p* ≤ 0.0001).

Regarding the respiratory parameters, it was found that hemin treatment led to a significant amelioration, characterized by a decrease in enhanced pause (Penh) (Figure 3(a)) and an increase in the respiratory frequency at 7th DAI (Figure 3(b)). Also, treatment with hemin led to a significant reduction of IFN-*γ* mRNA in lung and serum protein levels, IL-10 in serum, MCP-1 protein in lung tissue lysates, and serum and TNF in serum (Figures 3(c)–3(h)).

### 3.5. Induction of HO-1 Protects Alveolar Capillary Barrier

**Figure 4:**
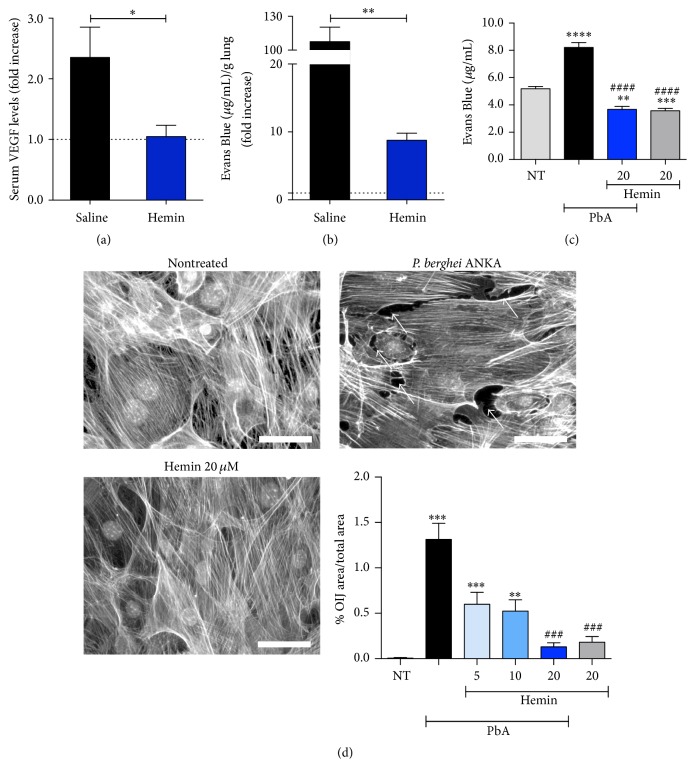
Induction of HO-1 defends the alveolar capillary barrier. (a) VEGF serum levels determined by ELISA (Mann–Whitney test, *n* = 10, ^*∗*^ *p* ≤ 0.05). (b) Lung endothelial permeability test (Mann–Whitney test, *n* = 10, ^*∗∗*^ *p* ≤ 0.01). (c) Permeability test of hemin treated (20 *μ*M) and nontreated (NT) PLMC (PbA: PLMC stimulated with PbA lysate). Significant difference versus nonstimulated is represented by “*∗*” and significant difference versus extract is represented by “#” (Kruskal–Wallis test with Dunn's multiple comparisons test, *n* = 24, ^*∗∗*^ *p* ≤ 0.01, ^*∗∗∗*^ *p* ≤ 0.001, ^*∗∗∗∗*^ *p* ≤ 0.0001, and ^####^ *p* ≤ 0.0001). (d) Pictures of PLMC by fluorescent microscopy (scale bars: 50 *μ*m). Ten to twenty pictures were taken for each culture and the most representative are presented in (d) (white arrows are pointing to opening interendothelial junctions (OIJ)). The graph (bottom right) represents the ratio of the area of OIJ per total area of each picture (Kruskal–Wallis test with Dunn's multiple comparisons test, *n* = 74, ^*∗∗*^ *p* ≤ 0.01, ^*∗∗∗*^ *p* ≤ 0.001, and ^###^ *p* ≤ 0.001). Hemin was given at 5, 10, and 20 *μ*M.

The treatment of PbA infected mice with hemin led to a decrease of VEGF serum levels (Figure 4(a)), a potent inducer of ALI/ARDS in PbA infected DBA/2 [10]. Also, hemin treated mice showed protection of the alveolar capillary barrier, which can be seen by a reduction in lung vascular permeability after Evans Blue administration and quantification (Figure 4(b)). In addition, we observed the same effect in PMLEC when they were hemin treated after being stimulated with PbA lysate for 3 hours, in a transwell plate (Figure 4(c)).

To understand a possible mechanism of HO-1 action in reducing pulmonary vascular permeability, PMLEC were treated with hemin in 5, 10, and 20 *μ*M, before PbA stimulus. The hemin action showed a significant reduction in the OIJ that are formed between the endothelial cells, when compared to nontreated cells (Figure 4(d)).

## 4. Discussion

The DBA/2 developed ALI/ARDS between the 7th and 12th DAI, with an incidence of 50% (average) when infected with PbA [10, 14]. Our results reinforce this finding, showing that the animals that began to die after the 7th DAI until the 12th DAI showed typical signs of ALI/ARDS, such as pleural effusion, alveolar edema and hemorrhage, alveolar wall damage, inflammatory infiltrates, and lower percentage of alveolar area. The parasitemia reaches about 20% when mice start to develop ALI/ARDS (7th DAI) and this value has a slight decrease from the 12th DAI onwards. Interestingly, human malaria-associated ALI/ARDS often occurs in patients who have already begun the antimalarial treatment, which also leads to a decrease in parasitemia [5].

HO-1 showed a protective role in experimental episodes of severe malaria, including cerebral malaria [15, 16, 31]. Moreover, treatment with HO-1 inhibitors such as zinc protoporphyrin IX (ZnPPIX) or tin protoporphyrin IX (SnPPIX) led to an enhancement of ALI/ARDS signals in the case of sepsis but had no effect in the cases of hyperoxia and experimental cerebral malaria [16, 24, 32]. However, our data showed that ALI/ARDS-developing mice had increased levels of mRNA and HO-1 protein, when compared with HP-developing mice and noninfected mice. Additionally, the levels of bilirubin, which represent an indirect measure of HO-1 activity [33], were higher in ALI/ARDS mice than in noninfected mice. Although it is not known whether this increase in HO-1 levels and HO-1 activity constitutes an effort to revert the ALI/ARDS phenotype in the infected mice, it was showed in previous publications that the treatment of* P. berghei* ANKA infected DBA/2 mice with CO (a product of HO-1) [34] and with a CO-releasing molecule [15] protected them against ALI/ARDS. Therefore, in order to clarify whether HO-1 is protective in this model, its induction was performed by hemin.

The treatment with the inducer of HO-1 (hemin) had beneficial effects in PbA infected DBA/2 mice and in PMLEC in contact with PbA lysate.* In vivo*, this treatment led to an improvement in the survival rate and in lung histology, with the absence of lung edema, higher alveolar area percentage, and the absence of pleural effusion at necropsy. Hemin treatment led to a decrease in parasitemia levels. It is an interesting effect, since hemin was observed to lyse malaria parasites in culture [35]. Additionally hemin treated mice infected with* Plasmodium chabaudi adami* also have a reduction in parasitemia [36]. Possibly, the antimalarial effect of hemin is also occurring in our model. Additionally, hemin induces HO-1, which reduces the oxidative stress in the host, which may increase its capacity of parasite clearance.

Hemin also led to an improvement of respiratory parameters, with a decrease of enhanced pause (Penh) and increase of respiratory frequency reinforcing the mitigation of clinical signals of ALI/ARDS, alongside an increase in survival rate. These respiratory parameters were previously used in a predictive model of malaria-associated ALI/ARDS in DBA/2 mice [14]. Additionally, there is evidence of Penh and respiratory frequency being altered in a model of lung injury by SO_2_ exposure in rats [37].

In addition to improvements in survival, lung histology, Penh, and respiratory frequency, the treatment with hemin led to a significant decrease in the levels of the proinflammatory cytokines IFN-*γ*, MCP-1, and TNF-*α*, which is in agreement with the survival increase and with improvement in lung parameters, as it was shown that TNF-*α* induced pulmonary vascular endothelial injury in an animal model [38]. Additionally, lung neutrophil accumulation and lung leak were abrogated in TNF-*α* knockout mice that were subjected to hemorrhagic shock [39]. IFN-*γ* was also considered to be a key contributor to ALI/ARDS in a hyperoxia mice model, where it was shown that this cytokine induced increases in lung alveolar permeability and neutrophil migration into lung air spaces [40]. Increased levels of MCP-1, chemokine involved in recruiting of monocytes, neutrophils, and lymphocytes, were found in pulmonary alveolar macrophages isolated from a rat model of immune complex-mediated acute inflammatory lung injury [41]. This shows that hemin reduced the levels of inflammatory cytokines that are important in ALI/ARDS, which corroborates with previous results showing that HO-1 induction by hemin has anti-inflammatory effects in models of sepsis and LPS induced ALI/ARDS in mice [25, 42]. As an anti-inflammatory cytokine, which was increased in hemin treated animals, which were protected against endotoxic shock [43] and whose effect in ALI/ARDS was shown to be protective [44], IL-10 was not expected to be decreased in hemin treated animals in our data. However, this cytokine was observed to be increased in mice that developed ALI/ARDS in our model before (unpublished data).

The treatment with hemin resulted in a reduction in serum levels of VEGF, a factor that promoted the development of ALI/ARDS in this model, in a previous study [10]. Furthermore, Siner and colleagues demonstrated that VEGF is a potent inducer of HO-1 enzyme and that the induction of this enzyme reduced the acute lung injury in mice caused by hyperoxia [32]. The same could be happening in our model of MA-ALI/ARDS, because the VEGF levels are increased on the 7th DAI, coinciding with an increase of HO-1 levels in serum and lungs. On the other hand, it was shown that treatment of PbA infected mice with VEGF prevented them from developing experimental cerebral malaria [45]. However, the contribution of VEGF to the increase of HO-1 might not occur soon enough to revert the MA-ALI/ARDS phenotype in our model. In this model, VEGF caused a deleterious effect, increasing the lung vascular permeability [10]. Additionally, it was shown that ALF492, a CO-releasing molecule, protected PbA infected DBA/2 from ALI and reduced VEGF levels of treated mice [15]. This corroborates our results that show a reduction in VEGF levels on hemin treated mice. However hemin treatment was also shown to have deleterious effects in a rat model of neuroinflammation, where it was responsible for increased levels of reactive oxygen species, brain tissue loss, microglial activation, and neuronal death [46].

In addition, improved pulmonary vascular permeability in hemin treated infected mice on the 7th DAI was observed. This corroborates previous studies that showed a reduction in the breakdown of alveolar capillary barriers in models of ALI/ARDS induced by LPS [42]. Additionally, in experimental cerebral malaria, the induction of HO-1 reduced the permeability of the blood brain barrier in PbA infected mice [16]. Also,* in vitro*, there was a reduction of the permeability of PMLEC stimulated with PbA and treated with hemin reinforcing the importance of HO-1 in this model.

Finally, PMLEC stimulated with PbA lysate and treated with different hemin concentrations led to a decrease in the opening of interendothelial junctions, indicating that hemin acts at the cellular level, protecting from the deleterious effect of the parasite in endothelial cells and consequently reducing the lung vascular permeability. This is in accordance with a previous publication where the presence of OIJ after acute lung injury initiation in an animal model was responsible for an increase in lung vascular permeability [47].

This data supports the hypothesis that the increase in HO-1 levels after the 7th DAI is a late effort to reverse the ALI/ARDS phenotype. Inducing HO-1 early in PbA infection has a protective effect against ALI/ARDS, making this enzyme a target for the prevention of MA-ALI/ARDS.

## Supplementary Material

The Supplementary Figure S1 shows the survival curve, percentages of parasitemia of P. berghei ANKA infected mice. Additionally, the Supplementary Figure S1 shows the necropsies, histological lung sections and alveolar area percentage of non-infected, ALI/ARDS and HP mice. The Supplementary Figure S2 shows the quantitative RT-PCR assays of lung tissue and mice serum ELISA of non-infected, treated with hemin and untreated mice.
