# Supplementary figures and images for: Association of Heme Oxygenase 1 with Lung Protection in Malaria-Associated ALI/ARDS
Source: Mediators Inflamm. 2016 Nov 15;2016:4158698. doi: 10.1155/2016/4158698 (PMC5126464; doi:10.1155/2016/4158698)

Fig S1

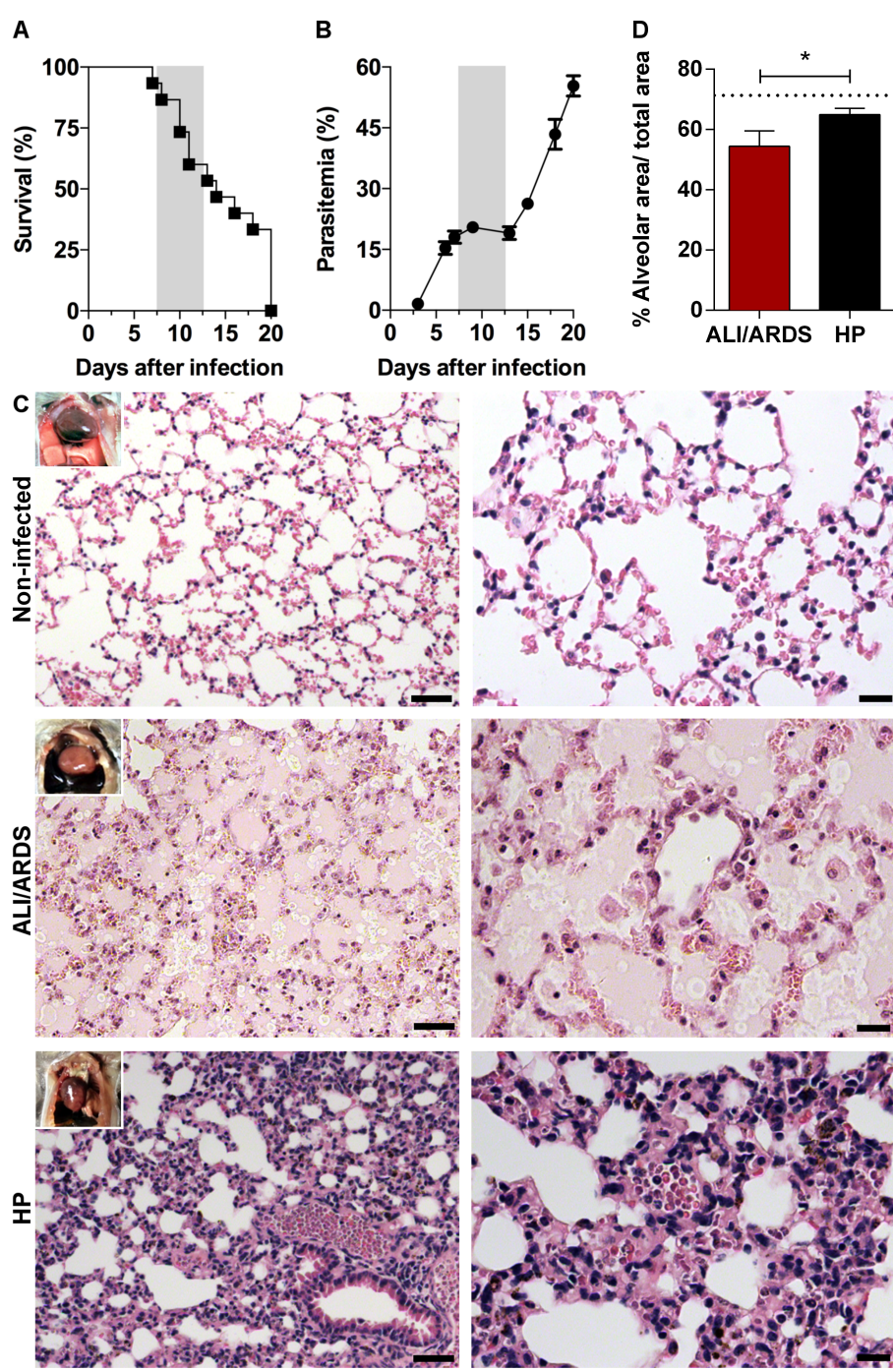

Fig S2

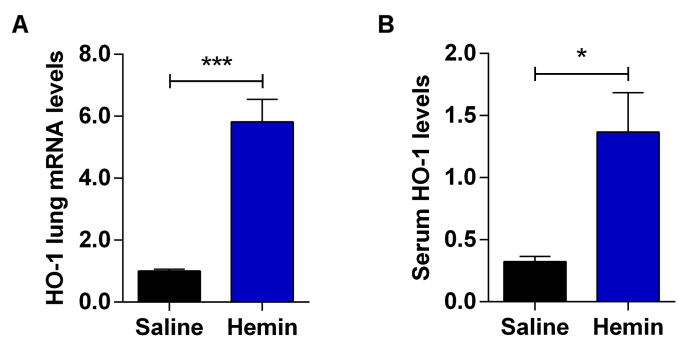

Supplement: Supplementary file 1 — The Supplementary Figure S1 shows the survival curve, percentages of parasitemia of P. berghei ANKA infected mice. Additionally, the Supplementary Figure S1 shows the necropsies, histological lung sections and alveolar area percentage of non-infected, ALI/ARDS and HP mice. The Supplementary Figure S2 shows the quantitative RT-PCR assays of lung tissue and mice serum ELISA of non-infected, treated with hemin and untreated mice. [file 4158698.f1.pdf]
